# Sensitive and rapid lateral-flow assay for early detection of subclinical mammary infection in dairy cows

**DOI:** 10.1038/s41598-020-68174-0

**Published:** 2020-07-07

**Authors:** Mohanned Naif Alhussien, Ajay Kumar Dang

**Affiliations:** 1 Animal Production Division, Agricultural College, Aleppo University, Aleppo, Syrian Arab Republic; 2 Lactation and Immuno-Physiology Laboratory, ICAR-National Dairy Research Institute, Karnal, Haryana 132 001 India

**Keywords:** Immunology, Innate immune cells

## Abstract

Detection of subclinical mastitis (SCM) in its initial stage can save great economic losses, improve milk quality and animal welfare. We have developed a semiquantitative lateral flow assay for the detection of SCM in dairy cows targeting myeloperoxidase (MPO) enzyme of milk neutrophils. A competitive immunoassay format was used, and colloidal gold nanoparticles (GNP) were prepared and used as a labelling agent. Monoclonal anti-MPO antibodies were used and assessed for its quality by enzyme-linked immunosorbent assay and dot blot. Conjugation method for GNP and anti-MPO antibodies was standardised, and the conjugate was placed over the conjugate pad. MPO coupled with a carrier protein (OVA) and the species-specific secondary antibodies were placed on test and control lines, respectively. The developed assay was verified with 75 milk samples collected from healthy, SCM and clinical mastitis cows. It displayed a high sensitivity as it could detect MPO as low as 1.5 ng/ml, an accuracy greater than 97% and showed no crossreactivity when crosschecked with other milk proteins. The developed assay can be used as an alternative for SCM diagnostic tests where lab structure are available for obtaining the lysate of milk SCC.

## Introduction

Mastitis is a complex and most expensive disease characterised by the inflammation of the udder of dairy animals which initiates an inflammatory immune reaction manifesting itself in either subclinical or clinical mastitis^1^. Mastitis is also a great food safety problem associated with chemical, physical, and bacteriological changes in the milk as well as condemnation of milk due to antibiotic residues which make it unfit for human consumption^2,3^. Subclinical mastitis (SCM) is the inflammation of the mammary gland that does not create visible changes in the milk^4^. Although the milk seems normal, cows with SCM produce less milk with poor quality including shorter shelf life of milk products and increased risk for milk hygiene since it contains various pathogenic organisms^5^. Moreover, if SCM is not diagnosed during early stage and proper management actions are not taken, it may develop into clinical mastitis (CM) and thus lead to substantial economic losses to the dairy sector^1^. Since SCM is not visible, it requires special diagnostic tests to be detected^6^. Although there are many diagnostic approaches for SCM, the somatic cell counts (SCC) and California mastitis test are still the most common and preferable methods^2,7^. These tests are mainly dependent on the total number of milk SCC, which primarily increase during SCM due to an increase in different leucocytes number^8^.

Healthy udder has SCC below 1.5 × 10^5^ cells/ml of milk, and this value increases several folds based on the level and stage of mammary infection^7,9^. The increase in SCC up to 2.5 × 10^5^ cells/ml does not necessarily indicate mammary infection. However, it may be attributed to various factors, including the productivity of the animal, lactation stage, parity, seasons, diurnal rhythm, etc.^2,7,10–12^. Neutrophils, the first line of cellular defense, form around 25% of the total leucocytes in normal milk and their number increase dramatically whenever there is an infection in the mammary tissues^8,13^. Therefore, diagnostic tests specifically detecting neutrophils related molecules can provide a more specific and reliable indicator of SCM than total SCC^7,14,15^.

Myeloperoxidase (MPO) is one of the major lysosomal enzymes stored in the azurophilic granules of neutrophils and plays a major role in innate immunity against invading microorganisms^16^. Therefore, MPO has been widely used as a novel biomarker for the diagnosis of multiple diseases in humans^17^. Twenty-five years ago, Cooray^14^ suggested the use of milk MPO as a marker for mammary infection in cattle and developed an ELISA for this purpose. They also developed a sandwich ELISA to estimate the concentrations of MPO in bovine serum and neutrophil extracts^18^. Recently, Depreester et al.^15^ developed a flow cytometric method for the determination of MPO in bovine blood neutrophils.

In the last decade, lateral flow assay (LFA) has gained more popularity and widely used in different fields of biology due to its simplicity, rapidity, cost-effective and suitability for field deployment^19^. This technique utilises antibody-antigen interactions which are visualised with the help of a labelling agent, i.e., colloidal gold to detect the presence or absence of the analyte of interest in the sample^20^. Several LFA have been developed in recent years for the diagnosis of different veterinary related diseases including Foot-and-Mouth Disease virus^21^, Contagious Agalactia^22^, African swine fever^23^, and brucellosis^24^. In the present study, monoclonal antibody against MPO was used for the detection of MPO in milk somatic cells by using a competitive format of LFA. This assay is highly accurate and allows the detection of SCM in dairy cattle without the need for expensive instruments which may help in early diagnosis and treatment of SCM.

## Materials and methods

### Chemicals and reagents

Bovine lactoferrin (Catalog No. L9507), serum albumin (Catalog No. A3608), lactoperoxidase (Catalog No. 61328), histone (Catalog No. 10223565001), trisodium citrate dehydrate (Catalog No. 6132-04-3), Goat anti-mouse IgG (Catalog No. A0168), Histopaque solutions 1077 (Catalog No. 10771) and 1119 (Catalog No. 11191), Xylenes (Catalog No. 214736), Methylene blue dye (Catalog No. M9140) were procured from Sigma-Aldrich (St. Louis, MO, USA). Concentrated hydrochloric acid (Catalog No. 137007) was purchased from Merck (Mumbai, India). Mouse monoclonal anti-MPO antibody (Catalog No. MAA601Bo21), OVA conjugated MPO (Catalog No. CPA601Bo21), and MPO sandwich ELISA (Catalog No. SEA601Bo) were procured from Cloud-Clone Corp (Houston, USA). DAB-peroxidase system (Catalog No. E733) was procured from Amresco (Solon, OH, USA). Mammalian protein Extraction Reagent, M-PER (Catalog No. 78501), phenylmethylsulfonyl fluoride, PMSF (Catalog No. 36978B), and TMB substrate (Catalogue No. N301) were procured from Thermo Scientific (Rockford, IL 61105, USA). HRP conjugated goat anti-mouse IgG (Catalogue No. 62114068001A) was purchased from Bangalore Genei (India).

Lateral flow assay pads including sample pad (Catalog No. FGB-R7L and FGB-R4); conjugate release matrix (Catalog No. PT-R5); adsorption pad (Catalog No. AP080: and AP045) and nitro cellular membrane [Catalog No. CNPF-SN12-L2-P25 (5 µm); CNPC-SS12-L2-P25 (10, 12, and 15 µm)] were procured from MDI (Ambala, India). Disposable cell culture plasticware, consumable and vacutainer tubes were procured from Coster, Sigma Aldrich (USA), Greiner Bio-One GmbH (Germany), MultiSkan GO, Thermo Scientific (Finland), and Reinfeld (Germany).

### Ethical permission

The experiment procedures were approved by the Institutional Animal Ethics Committee of Indian Council of Agricultural Research-National Dairy Research Institute (ICAR-NDRI) and were performed according to the guidelines and rules framed by the Committee for the Purpose of Control and Supervision of Experiments on Animals (CPCSEA), Government of India (Reg. No. 1705/GO/ac/13/CPCSEA, dated. 3/7/2013).

### Preparation of colloidal gold nanoparticles (GNPs)

The citrate reduction method was considered for the preparation of gold nanoparticles in the aqueous phase as reported by Liu and Lu^25^. In this method, the trisodium citrate is utilised for gold chloride reduction. Briefly, 99.5 ml Milli-Q water was added to a 250 ml double-neck flask and placed over a magnetic stirrer (Tarsons, USA). The flask was connected to the refluxing condenser, 0.5 ml of gold chloride solution (50) mM was added, and heating and stirring were initiated. When the solution starts refluxing, 10 ml of 38.8 mM tri-sodium citrate di-hydrate solution was added, followed by heating and stirring steps. After a deep red colour was formed, the content was allowed to reflux for 20 min. After that, the content was cooled to room temperature under constant stirring. The prepared GNPs were stored in a clean amber-coloured glass container at 4 °C without any preservatives.

### Physico-chemical characterisation of the prepared GNPs

The absorption spectra of GNPs was measured in the range of 700 to 300 nm (bandwidth = 0.5 nm) against Milli-Q water as blank using a Microplate reader (Multiskan Go, Thermo Scientific, Finland). Moreover, the storage stability of prepared GNPs was monitored by recording the absorption spectra at weekly intervals. Dynamic light scattering (DLS) technique was used to determine the average size and zeta potential of the prepared GNPs using DLS based instrument (ZS-90, Malvern, UK) as described by Saha et al.^26^.

### Verifying the quality of anti-MPO monoclonal antibodies

Quality of anti-MPO monoclonal antibody in term of sensitivity and specificity was checked by indirect ELISA. For this experiment, neutrophils were isolated from milk by the gradient density centrifugation method using Histopaque solutions 1077 and 1119 as described by Alhussien and Dang^13^. Extracts of milk lymphocyte were obtained by density gradient centrifugation on Percoll as described by Rivas et al.^27^. M-PER was added to lyse the collected neutrophils and lymphocytes, 0.5 mM of PMSF was added to the lysis buffer to avoid protein degradation. Wells of a polystyrene microtiter plate were coated with 100 µl of OVA conjugated MPO, neutrophil lysate, lymphocyte lysate, lactoperoxidase, lactoferrin and histone (2 μg/ml). After overnight incubation, washing and blocking steps were performed, the monoclonal MPO antibody in various concentration of 1,000, 750, 500, 250, 125, 60 (ng/100 µl) was used, and normal ELISA protocol was followed as described by Zehnacker et al.^28^. Finally, the absorbance was taken at 450 nm using a microplate reader (Multiskan Go, Thermo Scientific, Finland).

Mouse monoclonal anti-MPO antibody was also analysed in a dot blot assay for its specificity and sensitivity. Briefly, 4 µl of neutrophil lysate, OVA conjugated MPO, lymphocyte lysate, histone, lactoperoxidase and lactoferrin was spotted on nitrocellulose membrane and dried. The membrane was blocked with 5% BSA in TBST. The dot blot assay was carried out using the primary antibody (1:750 diluted with 3% BSA in TBST) and the HRP-conjugated secondary antibody (1:1,500). DAB-peroxidase system was employed to detect the signal, and the results were photographed using a Sony digital camera.

### Conjugation of GNPs with anti-MPO monoclonal antibody

Initially, different concentrations of the anti-MPO monoclonal antibody, viz. 0, 5, 10, 20, 40 and 80 µg/ml in borate buffer (0.1 M, pH 7.5) were tried, and aggregation experiment (using 100 μl of 10% aqueous sodium chloride solution) was carried out to check the optimum concentrations of anti-MPO monoclonal antibody needed for conjugation. The conjugation of anti-MPO monoclonal antibodies with GNPs was done following the protocol described by Thobhani et al.^29^. Briefly, 1 ml of colloidal gold solution was centrifuged (12,000 rpm, 20 min, 4 °C) and the GNPs pellet was suspended in borate buffer (1 ml, 0.1 M, pH 7.5) containing anti-MPO monoclonal antibody of 20 μg/ml. After incubation on Rotospin Test Tube Rotator (Tarson, India) for 30 min at 12 rpm, blocking was performed using 10% BSA in borate buffer (0.1 M, pH 7.5). The antibody–GNPs conjugate was then washed and suspended in borate buffer (250 μl, 0.1 M, pH 7.5) containing 0.1% BSA.

### Milk collection and classification using different SCM diagnostic techniques

Milk samples were collected from 75 Sahiwal and Karan Fries cows, maintained at Livestock Research Center, NDRI, Karnal. All the cows were multiparous, having similar body condition and milk yield (10–15 kg/day) and were in the early stage of lactation (days in milk < 90). The health status of mammary gland was checked using several diagnostic tests including California Mastitis Test (CMT), counting of milk SCC, and by measuring electrical conductivity (EC). The procedure of CMT was performed according to the manufacturer’s protocol. Briefly, 2 ml of milk sample was taken in the CMT paddle kit, and 2 ml of CMT reagent (DeLaval Pvt. Ltd, India) was added in each cup. The mixture was rotated for 30 s, and the result was recorded as 0 (negative, mixture remains unchanged), score 1 (trace or slight gel formation), score 2 (slight to distinct gel formation), and score 3 (distinct and strong gel formation). The EC of milk samples were determined by Lactoscan milk analyser (Milkotronic Ltd, Stara Zagora, Bulgaria). The counting of somatic cell in milk was carried out using a digital system of a lactoscan milk somatic cell counter (Milkotronic Ltd, Stara Zagora, Bulgaria) as described by Alhussien and Dang^7^. Counting of somatic cells in milk was crosschecked microscopically using 96% ethyl alcohol for fixation, Xylene to remove fat and Methylene Blue dye for staining. Milk SCC was counted microscopically at 40 ×, and the percentage of neutrophils was counted in 30 fields under oil immersion at 100 × (Olympus IX51 microscope) as described by Alhussien and Dang^13^. Neutrophil (%) = [no of neutrophils counted/total no of cells (macrophage, lymphocyte and neutrophils)] × 100.

### Quantification of MPO in milk neutrophils

Sandwich ELISA kit (Bovine specific) was used to estimate the concentration of MPO in milk neutrophils. Neutrophils were lysed using a bead beater (Unigenetics Instrument Pvt. Ltd., India). The minimum detectable dose of bovine MPO was 0.64 ng/ml, and the detection range of the assay was 1.56–100 ng/ml. The intra and inter-assay CV were 10% and 12%, respectively.

### Optimisation of lateral flow assay membranes and preparation of test strips

To optimise the ideal membrane components; various pore size of nitrocellulose membranes (5, 10, 12 and 15 µm), two different sample pads (FGB-R7L and FGB-R4) and two different thickness of adsorption pad (AP080: 0.9 mm thickness, AP045: 0.45 mm thickness) were used. One conjugate pad (PT-R5) was employed during all the tests. Initially, we tried both sandwich and competitive LFA formats, and finally, we proceeded with the competitive format as the results were more satisfactory. OVA conjugated MPO antigen (0.25 µg/µl, in 0.1 M PBS, pH 7.4) and secondary antibody (goat anti-mouse IgG-antibody) were printed on the test and control lines of the nitrocellulose membrane, respectively, using an LFA printer (LPM-02, MDI, Ambala, India) at 2 µl/cm of strip width. The printed membrane was dried under controlled atmospheric condition and used for the construction of the LFA strip. The sticky layers on both sides of the backing card of nitrocellulose membrane were exposed, and the conjugate pad was pasted with 2 mm overlaps the nitrocellulose membrane at the appropriate side. The conjugation pad was then overlapped by sample pad, and the adsorbent pad was pasted to the opposite side of the nitrocellulose membrane. The assembled strip was cut into strips of uniform width (5 mm) using a strip cutter. GNPs conjugated with anti-MPO antibody (5 µl) were deposited on the conjugation pad and allowed to dry at 30 °C. The strips were stored in an air-tight plastic box at room temperature.

### Detection of subclinical mastitis (myeloperoxidase) in milk using LFA

Around 50 ml of milk sample of each cow was centrifuged, and the obtained cell pellet was taken. Cell lysate was obtained either by M-PER or bead beater (Unigenetics Instrument Pvt. Ltd., India) and 200 µl of lysis of somatic cell pellet was used to run the test. Initial testing trials were performed with an aqueous solution of OVA conjugated-MPO (at different concentration), then it was tested with lysis of somatic cell pellet from healthy and mastitis samples. The appearance of two lines (i.e. at control as well as test line) indicated that the MPO analyte in the sample is either below the detection limit or absent. The appearance of one line (i.e. at control line) indicated that the MPO analyte was above the detection limit.

### Sensitivity, specificity and storage stability of the developed assay

To check the sensitivity of the developed test, 75 milk samples were collected from Karan Fries (crossbred) and Sahiwal (indigenous) cows. The milk samples were checked by several techniques to evaluate the health status of mammary gland and classified as healthy (H; CMT score = 0, SCC < 1.5 × 10^5^ cells/ml, EC = 5.90 mS/cm), early stage of subclinical mastitis (SCM-1; CMT score = 1, SCC = 1.5–3.5 × 10^5^ cells/ml, EC = 6.10 mS/cm), advanced stage of subclinical mastitis (SCM-2; CMT score = 2, SCC = 3.5–5.5 × 10^5^ cells/ml, EC = 6.40 mS/cm) and clinical mastitis (CM; CMT score = 3, SCC > 5.5 × 10^5^ cells/ml, EC = 7.20 mS/cm)^3, 7, 9, 13^. Various clinical symptoms were considered during the selection of cows with clinical mastitis including swelling, redness and pain in the infected quarter, depressed appetite, fever and altered milk with visibly abnormal signs such as colour, consistency, blood in milk, etc. The same results were crosschecked by LFA. The results were classified as correct positive (CP) when the LFA test was showing subclinical or clinical sample correctly. False negatives (FN) when showing mastitis sample as healthy and false positives (FP) when showing a healthy sample as mastitis. Correct negatives (CN) were considered when the test was showing that milk sample free from inflammation correctly. To calculate the visual limit of detection (LOD), Stock solution (1.0 mg/ml) of OVA conjugated-MPO antigen was used to prepare different concentrations, i.e., 0, 1, 1.5, 2, 2.5, 3, 5, 10, 20, 30, 50 and 100 (ng/ml) in borate buffer. The test was run with different concentrations of OVA conjugated-MPO, and the LOD was recorded.

The specificity of the developed assay was tested in a crossreactivity study with bovine lactoferrin, histone, and lactoperoxidase which are major proteins released by neutrophil during an inflammatory response. Moreover, extracts of milk lymphocyte were used in the crossreactivity study. The functionality of the LFA strips during storage was tested at fortnightly intervals. The strips were tested with borate buffer, and lysate of milk somatic cell and the results were noted.

### Statistical analysis

The data were analysed by repeated-measures ANOVA using the mixed procedure of SAS (Proc Mixed, SAS Institute Inc., Cary, NC, USA, version 9.1). The pairwise comparison was performed using a multiple comparison test (Tukey). *P* values less than 0.05 were considered as statistically significant.

## Results and discussion

### Quality determination of the anti-MPO monoclonal antibody

**Figure 1:**
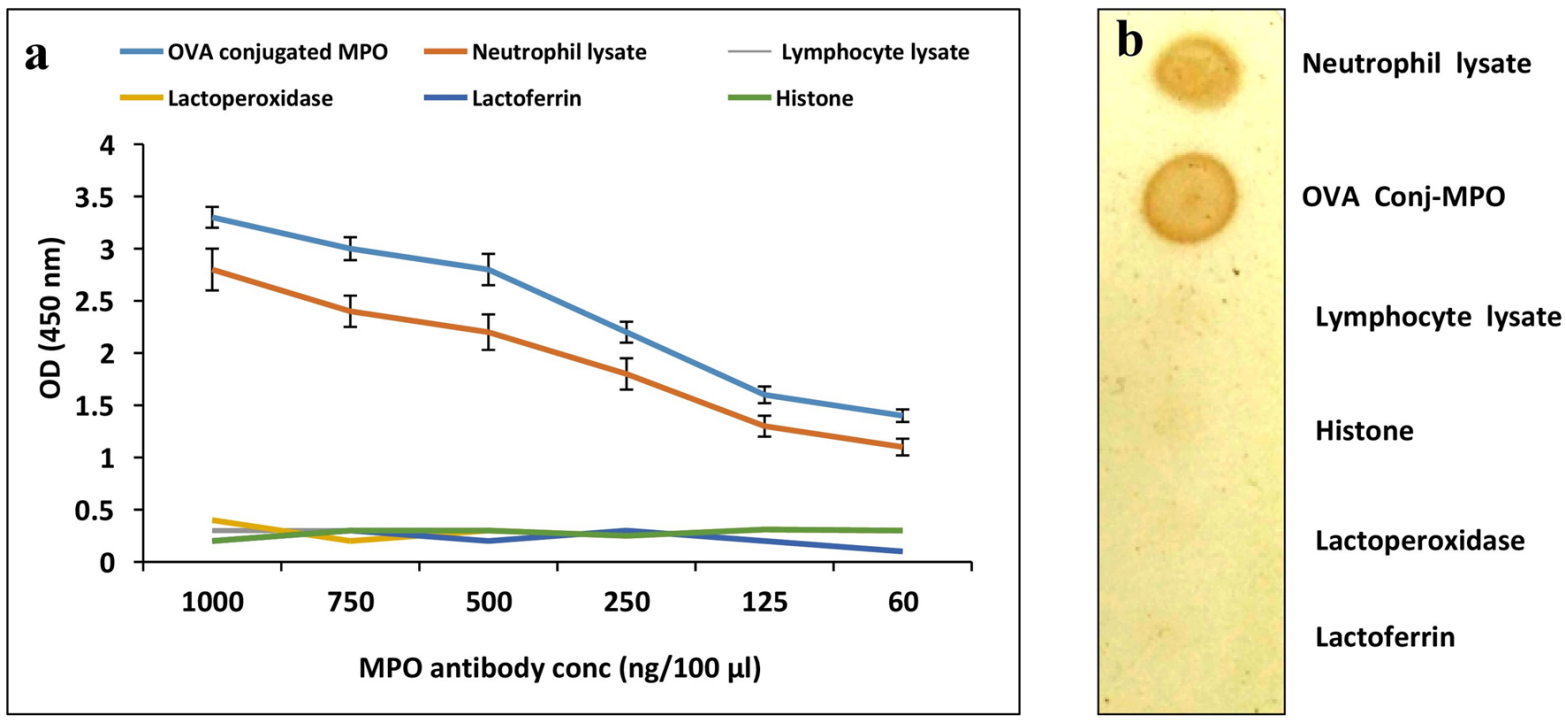
Absorbance of different concentrations of anti-MPO monoclonal antibody generated against OVA conjugated MPO, neutrophil lysate, lymphocyte lysate, lactoperoxidase, lactoferrin and histone (**a**). Reactivity and specificity of mouse monoclonal anti-MPO antibody with neutrophil lysate, OVA conjugated MPO, and other proteins in dot blot using DAB system (**b**).

The commercially available mouse anti-MPO monoclonal antibody was used for the diagnosis of SCM in dairy cows targeting MPO. Quality of anti-MPO monoclonal antibody in term of sensitivity and specificity was verified by indirect ELISA (Fig. 1a). OVA conjugated MPO, neutrophil lysate, lymphocyte lysate, neutrophil histone and other enzymes (lactoperoxidase, lactoferrin) that are present abundantly in milk during inflammatory conditions were studied for their interaction with different concentrations of anti-MPO monoclonal antibody. The absorbance value at 450 nm decreased (*P* < 0.05) at a higher dilution of the antibody, and it was higher (*P* < 0.05) for the pure form of MPO as compared to the neutrophil lysate. Moreover, there was no reaction or crossreactivity against lymphocyte lysate, lactoperoxidase, lactoferrin and histone. The results were also verified by dot blot, and similar findings to that of ELISA were obtained (Fig. 1b). This indicates that the antibody is highly specific for its target antigen and don’t crossreact with other proteins that present in milk during udder infection.

### Preparation and physico-chemical characterisation of gold nanoparticle (GNP)

**Figure 2:**
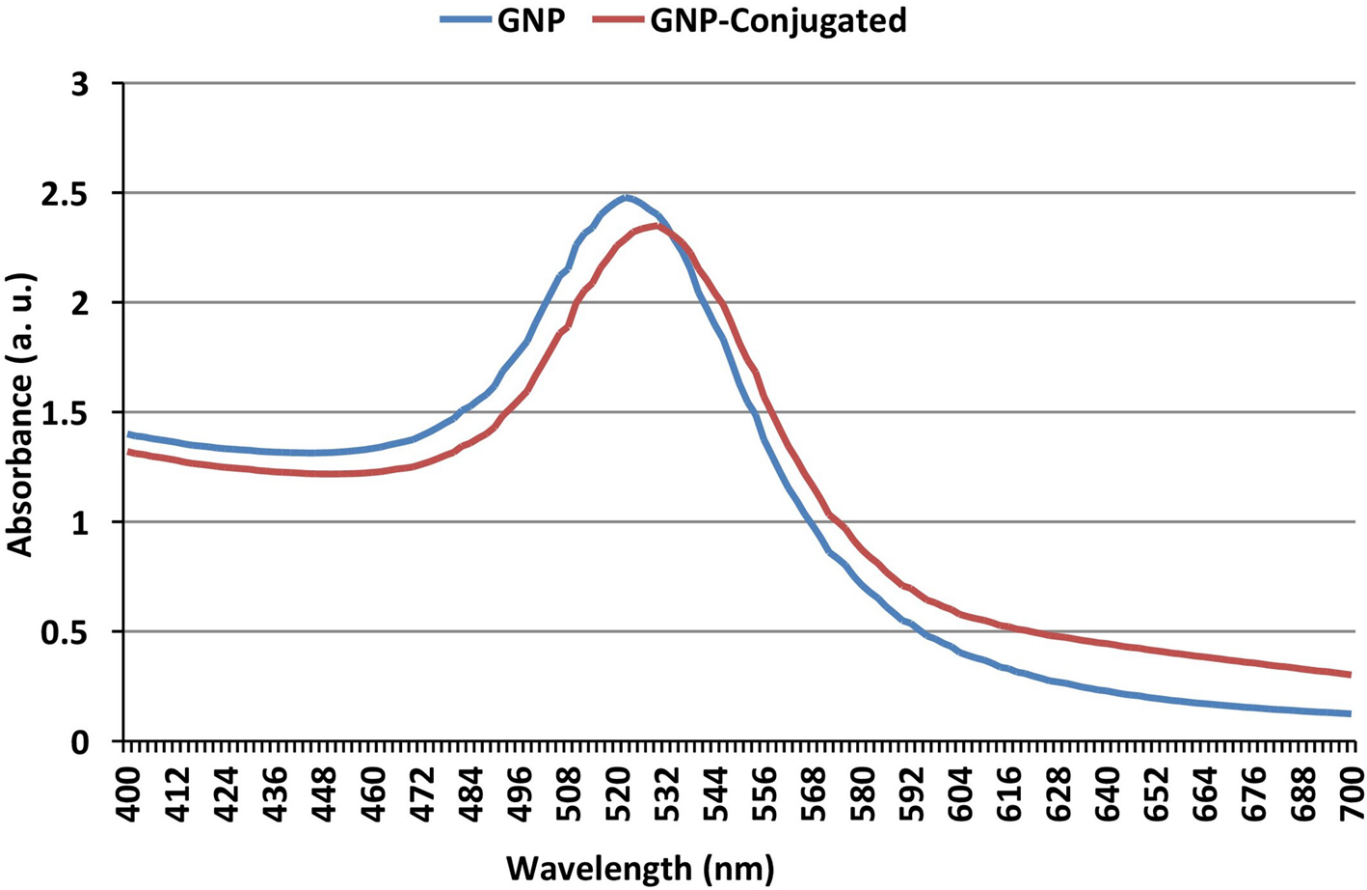
UV light scanning of both GNP and GNP conjugated with anti-MPO monoclonal antibody.

Colloidal gold nanoparticles (GNPs) are highly recommended as a label in LFA. GNPs were prepared in the present study using the liquid chemical method by reduction of chloroauric acid utilising sodium citrate as a reducing agent. The quality of synthesised GNPs was evaluated using various techniques before their application in the assay. GNP usually shows strong absorption of electromagnetic waves in the visible range because of plasmon resonance which is caused by the collective oscillations of the conduction electrons of GNP when exposed to light irradiation^30^. In the current study, the prepared GNPs exhibited a plasmon resonance peak at 524 nm (Fig. 2). Ideal size of GNPs is essential for successful development of LFA and can be controlled by altering the ratio of gold chloride and the reducing agent. The size of the prepared GNPs was assessed with dynamic light scattering (DLS)-based particle size analyser. The DLS technique measures the hydrodynamic size of nanoparticles in a solution. Since the intensity of the scattered light is directly proportional to the six power of the size of particle, larger particles display a greater signal than a smaller one^29^. It is a rapid measure that identifies the presence/absence of aggregates in the GNP with low volume needed.

**Figure 3:**
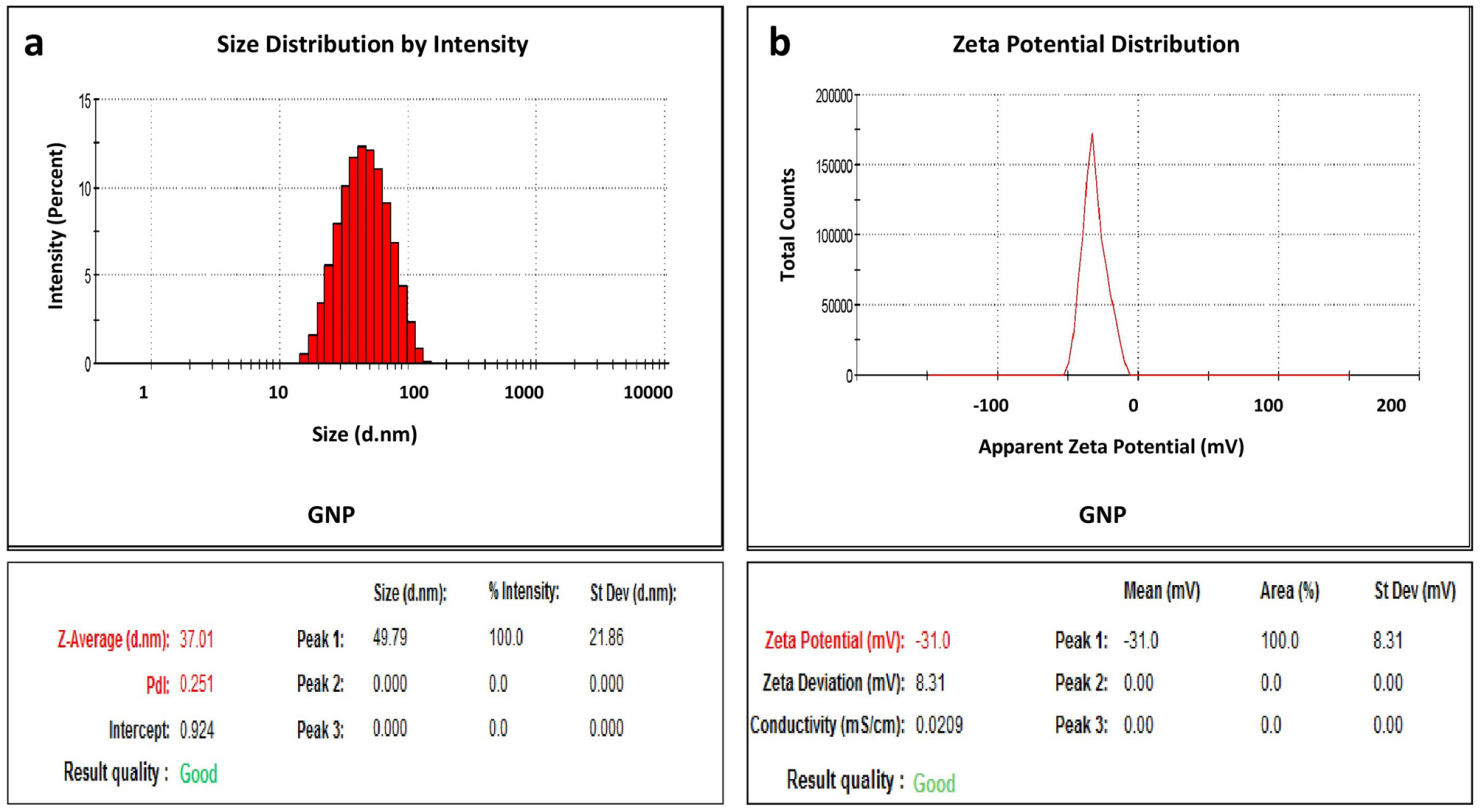
Average size distribution (% Intensity) of GNP (**a**). Apparent zeta potential (mV) of GNP (**b**).

The size of GNPs is presented in the form of a histogram to analyse the size distribution (Fig. 3a). Interestingly, 100% (intensity) of the prepared GNPs had an average size of 37.01 nm with a peak at 524 nm. Thobhani et al.^29^ synthesised GNP and reported average size of 40 nm and maximum absorption of 524 nm. Similarly, Nara et al.^31^ reported the average size of 36 nm and maximum absorption at 522 nm. Zeta potential is widely used to evaluate the stability of the colloidal system. Nara et al.^31^ used citrate reduction method for GNP preparation and reported zeta potential of − 44.1 mV which is highly stable as particles with zeta potential > + 30 mV and < − 30 mV are normally considered stable in suspension. In our study, the prepared GNPs had a zeta potential of − 31 mV (Fig. 3b), which is within the recommended range. The prepared GNP was stable for around 4 months at 4 °C. The stability of GNP during storage was evaluated through spectral observation at weekly intervals (data not shown). There was no change in the spectral peak up to 4 months, indicating the stability of the prepared GNPs.

### Antibody–GNP conjugation

**Figure 4:**
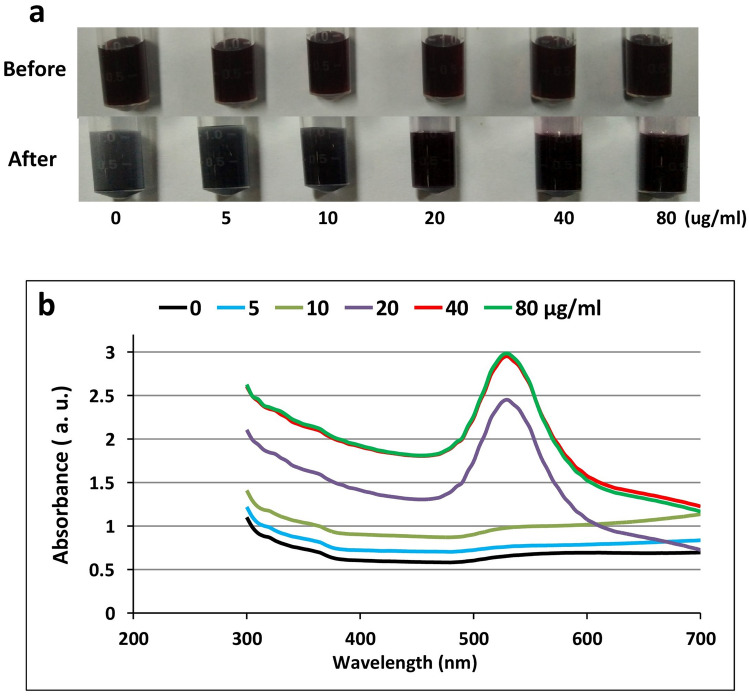
Optimization of anti-MPO antibody (Conc. 0–80 μg/ml) for the conjugation of GNP. The aggregation of the GNP-conjugated to MPO antibody before and after the addition of 10% NaCl (**a**). The absorbance of the GNP-conjugated to MPO antibody at different concentrations (**b**).

LFA is an immunogenic technique in which antibody against the target antigen is conjugated to colloidal gold and the stability of the formed conjugate is essential for successful development of the assay. A stable conjugation between GNPs and anti-MPO monoclonal antibody was successfully formed following a protocol described by Thobhani et al.^29^. The conjugation between antibody and colloidal gold is facilitated through electrostatic and hydrophobic interactions^32^. Colloidal gold has a negative charge due to the coated citrate ions on its surface. It displays a natural affinity for protein (antibody) to form a stable conjugate when optimum conditions are provided. To achieve that, the prepared GNPs were dissolved in borate buffer (pH 7.5) containing the optimum concentration of antibodies. Coating the colloidal surfaces with protein molecules, viz. antibody could provide stability and protect it from NaCl^33^. The optimum concentration of anti-MPO monoclonal antibody (20 μg/ml of GNPs) was determined by adding 100 µl NaCl and observing the formation of aggregates (Fig. 4). Multiple techniques confirmed successful conjugation of GNPs to antibody. UV light scanning of GNPs showed a peak at 524 nm, whereas the GNPs conjugated with antibody displayed shifting in the peak to 530 nm (Fig. 2). The average size of GNPs also shifted from 37 to 73 nm (data not shown) indicating successful conjugation of anti-MPO antibody with the GNPs*.*

### Estimation of MPO concentration and neutrophils percentage

MPO is one of the effector molecules released by activated neutrophils and have antimicrobial properties essential for the innate defence mechanism against the invading pathogens. Moreover, it is essential for the transformation of hydrogen peroxide (H_2_O_2_) into hypochlorous acid (HOCl) during the neutrophil’s respiratory burst which is highly efficient in killing the invading microorganisms, and also causes huge cytotoxic damage to the host tissue^15,34^. The MPO enzyme is recognised as an inflammation biomarker in human medicine and is used as an important diagnostic tool for bacterial infections^35^, diagnosis of the wound^36^ and the urinary tract infections^37^. Although MPO enzymes have been recognised as an important molecule that can be used efficiently to diagnose various bacterial infections in bovine, this is the first effort to develop LFA for the diagnosis of subclinical mastitis using this molecule.

**Figure 5:**
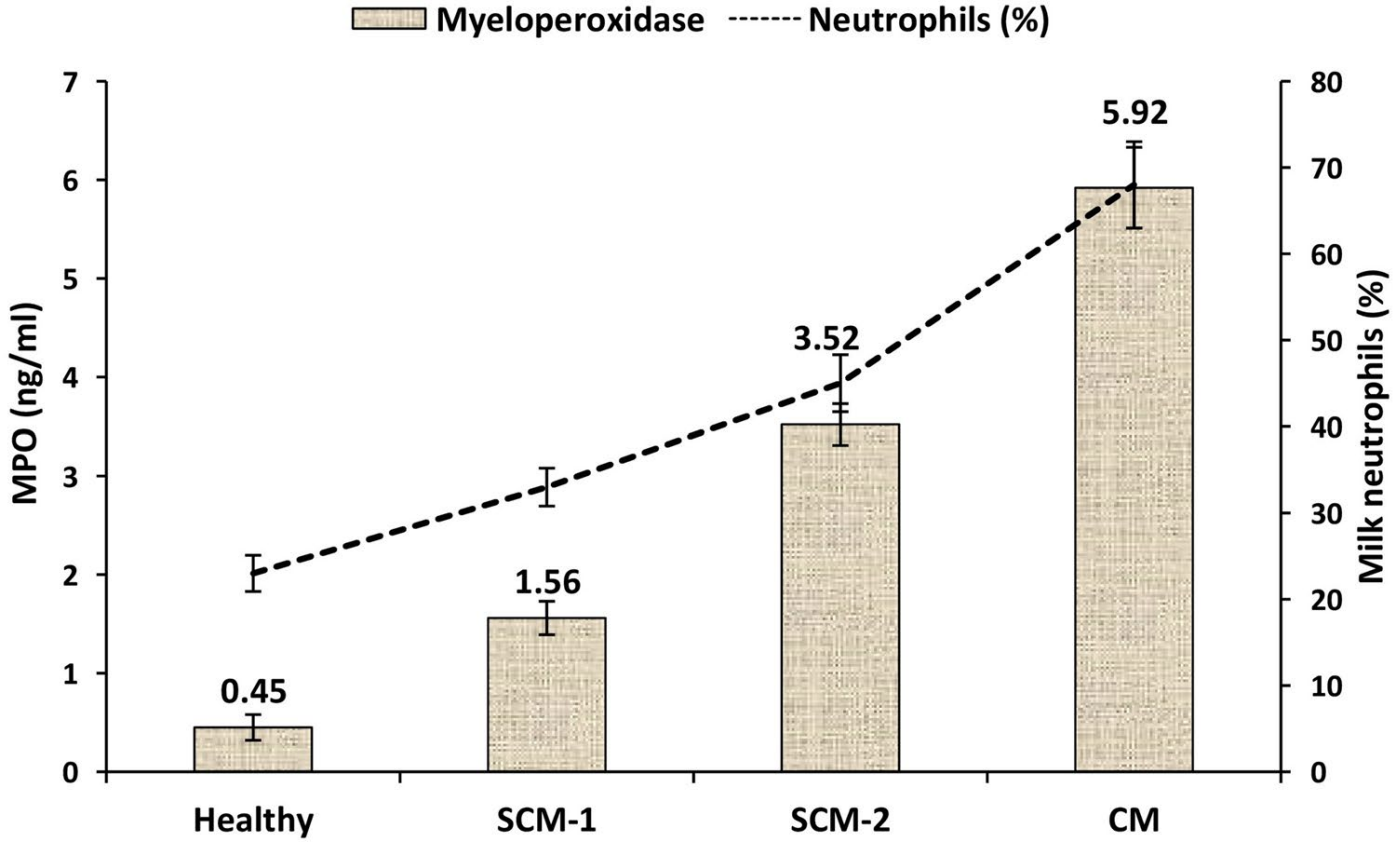
Myeloperoxidase concentrations in milk neutrophil lysate and neutrophil percentage of healthy (CMT score = 0, SCC < 1.5 × 10^5^ cells/ml, EC = 5.90 mS/cm), subclinical mastitis-1 (SCM-1; CMT score = 1, SCC = 1.5–3.5 × 10^5^ cells/ml, EC = 6.10 mS/cm), subclinical mastitis-2 (SCM-2; CMT score = 2, SCC = 3.5–5.5 × 10^5^ cells/ml, EC = 6.40 mS/cm), and clinical mastitis (CM; CMT score = 3, SCC > 5.5 × 10^5^ cells/ml, EC = 7.20 mS/cm) cows.

The percentage of milk neutrophils increased (*P* < 0.05) from around 25% in healthy milk samples to about 33–45% in SCM samples and reached up to 70% in the milk samples collected from mastitis cows (Fig. 5). Similarly, the concentrations of MPO followed a similar trend of milk neutrophils in which it was lowest in healthy cows and increased (*P* < 0.05) with the elevated somatic cell counts to attend maximum values in mastitis cows (Fig. 5). It is already well known that the major and the most common leukocyte cells appearing in milk during mammary infection are neutrophils^8,9^. Moreover, the concentration of MPO in bovine neutrophil has been reported to increase in association with the increased neutrophil number during calving and mammary infection^14,38,39^. The presence of up to 2.5 × 10^5^ somatic cells per ml of milk does not necessarily indicate SCM; however, it may be attributed to the productivity of the animal, lactation stage, parity, seasons, diurnal rhythm, etc^11,12^. Although LFA strip has been developed for the detection of *Streptococcus uberis* in raw milk, this test is limited to mammary infections associated with *S. uberis*^40^. The concentration of myeloperoxidase increases in parallel with the elevation in the number of neutrophils mainly during inflammatory condition as observed in the present study^16,17^. Therefore, neutrophil myeloperoxidase was targeted in this study as it can provide a more specific and reliable indicator of SCM than total SCC present in milk^14^.

### Detection of myeloperoxidase using a competitive format of LFA

**Figure 6:**
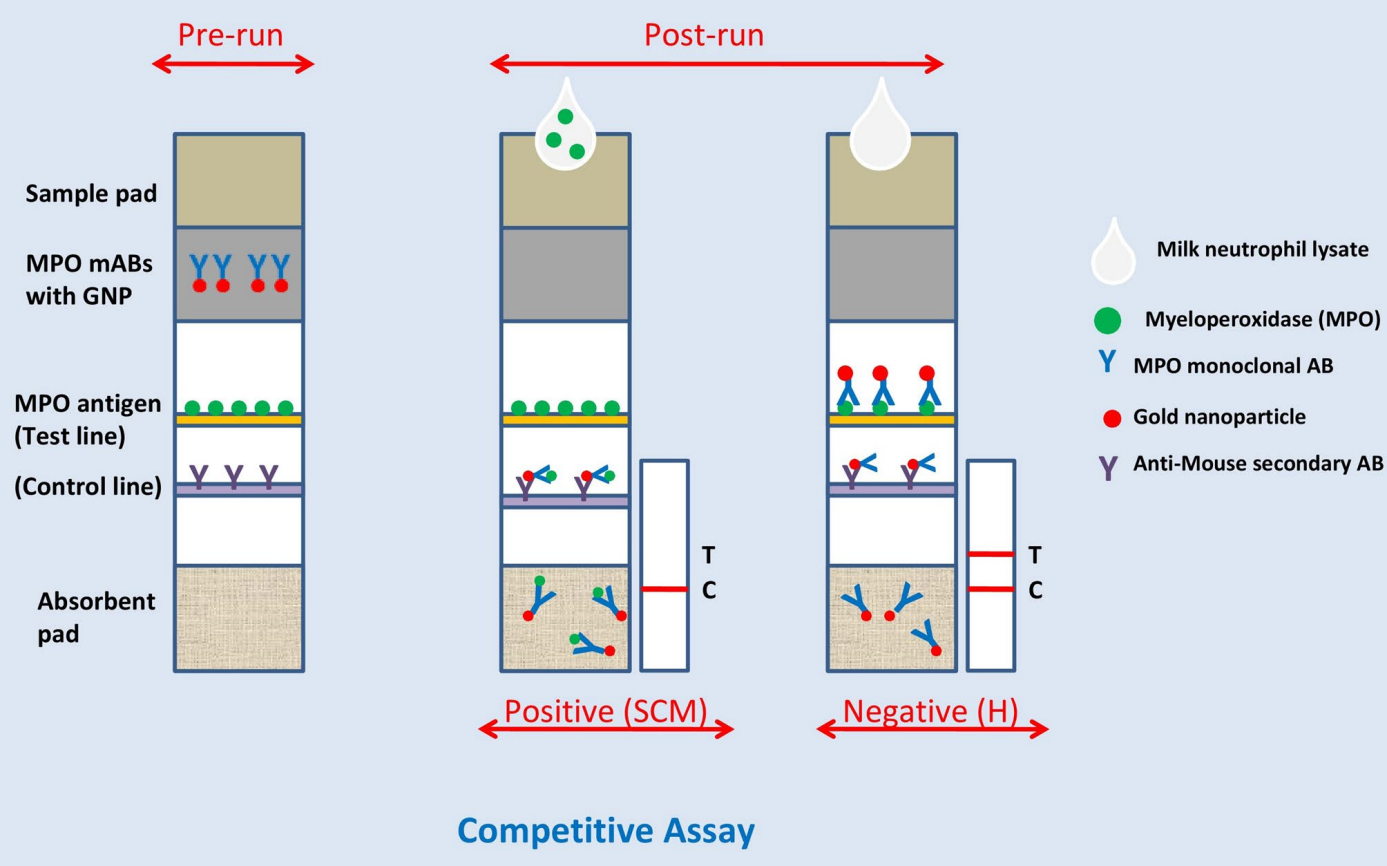
Components of lateral flow assay and working principle for MPO detection in milk neutrophils.

**Figure 7:**
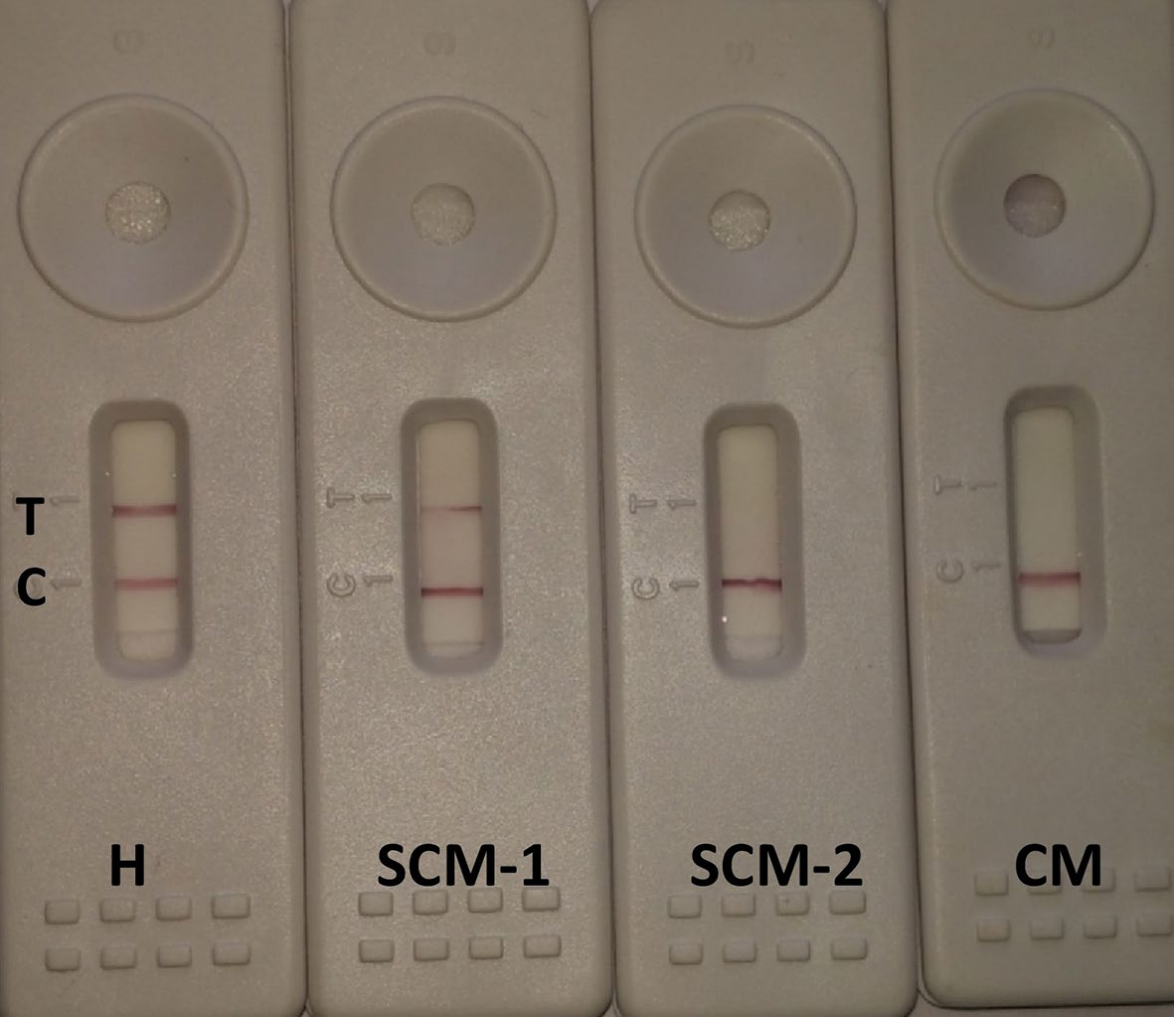
Validation of the developed LFA test in milk samples. Milk samples were classified to healthy (CMT score = 0, SCC < 1.5 × 105 cells/ml, EC = 5.90 mS/cm), subclinical mastitis-1 (SCM-1; CMT score = 1, SCC = 1.5–3.5 × 105 cells/ml, EC = 6.10 mS/cm), subclinical mastitis-2 (SCM-2; CMT score = 2, SCC = 3.5–5.5 × 105 cells/ml, EC = 6.40 mS/cm), and clinical mastitis (CM; CMT score = 3, SCC > 5.5 × 10^5^ cells/ml, EC = 7.20 mS/cm).

After a preliminary study, the selection of the LFA components were optimised as follows: Nitrocellulose membrane (CNPF-SN12-L2- H50) with pore size of 10 μm and wicking rate of 180 s for an area of 4 cm^2^; sample pad (FGB-R7L); conjugate pad (PT-R5); and absorbent pad (AP080: 0.9 mm thickness) were taken. The components of the developed lateral flow assay and the working principle for myeloperoxidase detection in the lysate of milk neutrophils are shown in Fig. 6. GNPs conjugated with anti-MPO monoclonal antibodies were deposited on the conjugation pad and dried at room temperature for 10 min. MPO antigen and species-specific secondary antibodies were drawn on test and control lines, respectively. Milk sample was centrifuged to obtain the somatic cells which were lysed using lysis buffer. LFA can be easily performed by individuals who are not even trained in chemical analysis. The developed LFA test can detect SCM efficiently in fresh milk samples as this assay is dependent on the reaction of the first line of cellular defense against mammary pathogens, i.e., neutrophils and not based on counting of total SCC in milk which shows great variability. However obtaining the extract of somatic cells for running the assay requires some basic lab facilities, which is the only limitation in this assay and may reduce its efficiency for screening a large number of lactating animals under farm conditions. On-farm level, the milk can be kept in standing position for 30 min, and the pellet settled at the bottom of the tube can be mixed with any cell detergent (SDS). Around 200 µl of the mixture (somatic cells and detergent) can be used to run the test, and results can be obtained within 5 min. After neutrophil lysate is placed on the sample pad, GNPs conjugated with anti-MPO antibodies starts moving through capillary action across the membrane. In this form of LFA, there is a competitive reaction between the free MPO in the sample (lysate of milk neutrophils), and the OVA conjugated MPO coated at test line for a limited amount of GNPs conjugated with MPO antibodies. Moreover, the intensity of the signal of red colour at the test line is inversely proportional to the concentration of MPO in the sample and the higher concentration of MPO in the tested sample, the weaker signal at the test line and vice-versa. If MPO is absent or present below detection limit (< 1.5 ng/ml) in the sample, the MPO coated at test line would interact with the gold-labelled anti-MPO antibodies and thus strong signal of red colour appears at the test line indicating that the cow is healthy and free from any udder infection (Fig. 7). When the MPO antigen presents in the sample in medium quantity (1–3 ng/ml), some MPO antibody will remain free and bind with MPO antigen at the test line and faded line can be seen at the test line indicating that the cow is in the initial stage of subclinical mastitis (SCM1). When MPO is present in high quantity (> 3 ng/ml), the antigen in the sample binds to the MPO antibody conjugated with gold, and it prevents any further interaction between the antibody and the MPO antigen at the test line and only control line can be seen indicating that the cow suffering from advanced form of SCM (Fig. 7). In competitive formats of LFA, the amount of GNP conjugated with anti-MPO antibodies placed at the conjugation pad is crucial and must be same in all strips. In our study, it was observed that 5 μl of GNPs conjugated to anti-MPO antibodies was sufficient and was maintained throughout the study. The time needed for running the test was 5 min, and test and control lines were found to be stable for a long time.

### Sensitivity and visual limit of detection of the lateral flow strip

**Figure 8:**
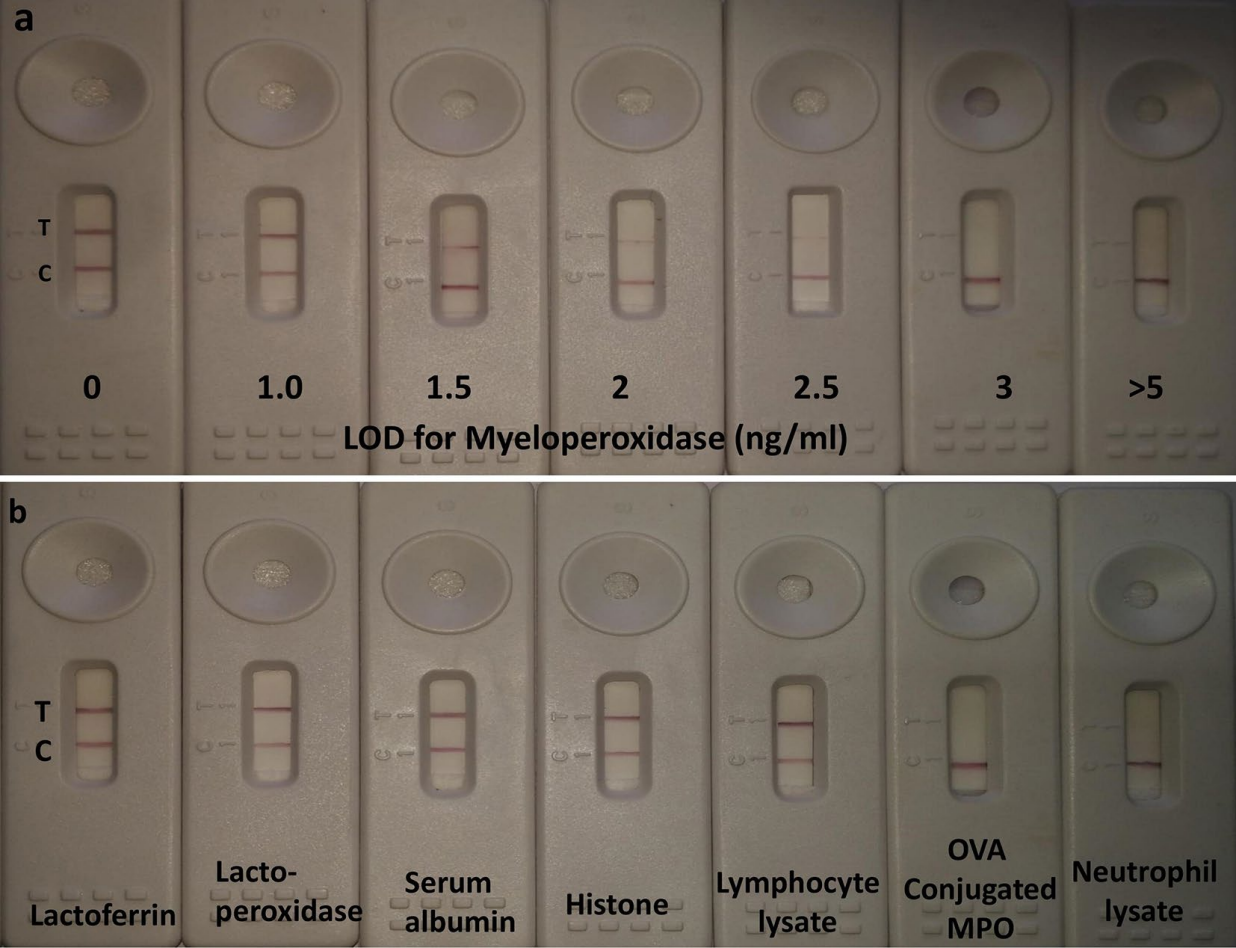
Visual limit of detection (LOD) for myeloperoxidase (**a**). Crossreactivity study for the developed assay (**b**). It shows from left to right; lactoferrin, lactoperoxidase, serum albumin, histone, lymphocyte lysate, OVA conjugated MPO and neutrophil lysate.

To check the sensitivity of the developed test, 75 milk samples were collected and evaluated firstly by several techniques (CMT, SCC, EC ) and then by using LFA. The results were classified as CP, FN, FP and CN. It was found that there is a 100% sensitivity for healthy samples having lower levels of MPO as well as an advanced stage of mastitis and mastitis sample with higher levels of MPO (Table 1). However, some false-negative results were observed in 2 samples that were classified as SCM-1 based on other SCM diagnostic tests and the sensitivity for SCM-1 samples was 90%. The milk samples were collected again from these two cows in the subsequent two days, and all the tests were repeated. It was revealed that the cows are healthy according to LFA and other SCM diagnostic tests including somatic cell counter as the SCC was below 1.5 × 10^5^ cells/ml. The overall sensitivity of the test was 97% (Table 1). There are many factors that can cause an increase in SCC up to 2.5 × 10^5^ cells/ml without the present of mammary infections^2,7,10–12^. Therefore, the FN results of LFA in the present study might be CN, and the developed LFA is more efficient in detecting the early stage of SCM (SCM-1) than the traditional somatic cell counter which still has to be verified with a large number of samples. Our results are in agreement with Cooray^14^, who suggested the use of milk MPO as a marker for mammary infection in cattle and developed an ELISA for this purpose. The same group developed a sandwich ELISA to estimate the concentrations of MPO in bovine serum and neutrophil extracts^18^. We extended the test to find out the visual limit of detection (LOD); it was found that it could detect as low as 1.5 ng of MPO in borate buffer (Fig. 8a) which is similar to the detection level of MPO observed in the neutrophil lysate (Figs. 5, 7).

**Table 1:** Validation of the developed LFA test in milk samples.

Groups	No. of sample tested	CP	FN	FP	CN	Sensitivity
Healthy	30				30	100
SCM-1	20	18	2			90
SCM-2	15	15				100
CM	10	10				100
Total	75	43	2		30	97

Milk samples were classified to healthy (CMT score = 0, SCC < 1.5 × 10^5^ cells/ml, EC = 5.90 mS/cm), subclinical mastitis-1 (SCM-1; CMT score = 1, SCC = 1.5–3.5 × 10^5^ cells/ml, EC = 6.10 mS/cm), subclinical mastitis-2 (SCM-2; CMT score = 2, SCC = 3.5–5.5 × 10^5^ cells/ml, EC = 6.40 mS/cm), and clinical mastitis (CM; CMT score = 3, SCC > 5.5 × 10^5^ cells/ml, EC = 7.20 mS/cm).

*CP* correct positive, *FN* false negatives, *FP* false positives, *CN* correct negatives.

### Crossreactivity study

The developed LFA shows no evidence of crossreactivity with various components present in milk in association with udder infection (lactoferrin, lactoperoxidase, serum albumin, histone and lymphocyte lysate) (Fig. 8b). Our results are similar to those observed by Cooray^14^ in milk. Hence; the developed LFA test exclusively detects MPO with high specificity.

### Conclusions

The developed assay is very sensitive, accurate and showed no crossreactivity with other milk proteins. The visual limit of detection of MPO is 1.5 ng/ml, and the results can be obtained within 5 min and visualized by eyes. Unfortunately, the developed test in its present form is not suitable for on-farm use as the extract of the somatic cells is needed to run the test. However, it can be used as an alternative for cow-side test of SCM detection where lab facilities are available for obtaining lysate of milk SCC.
